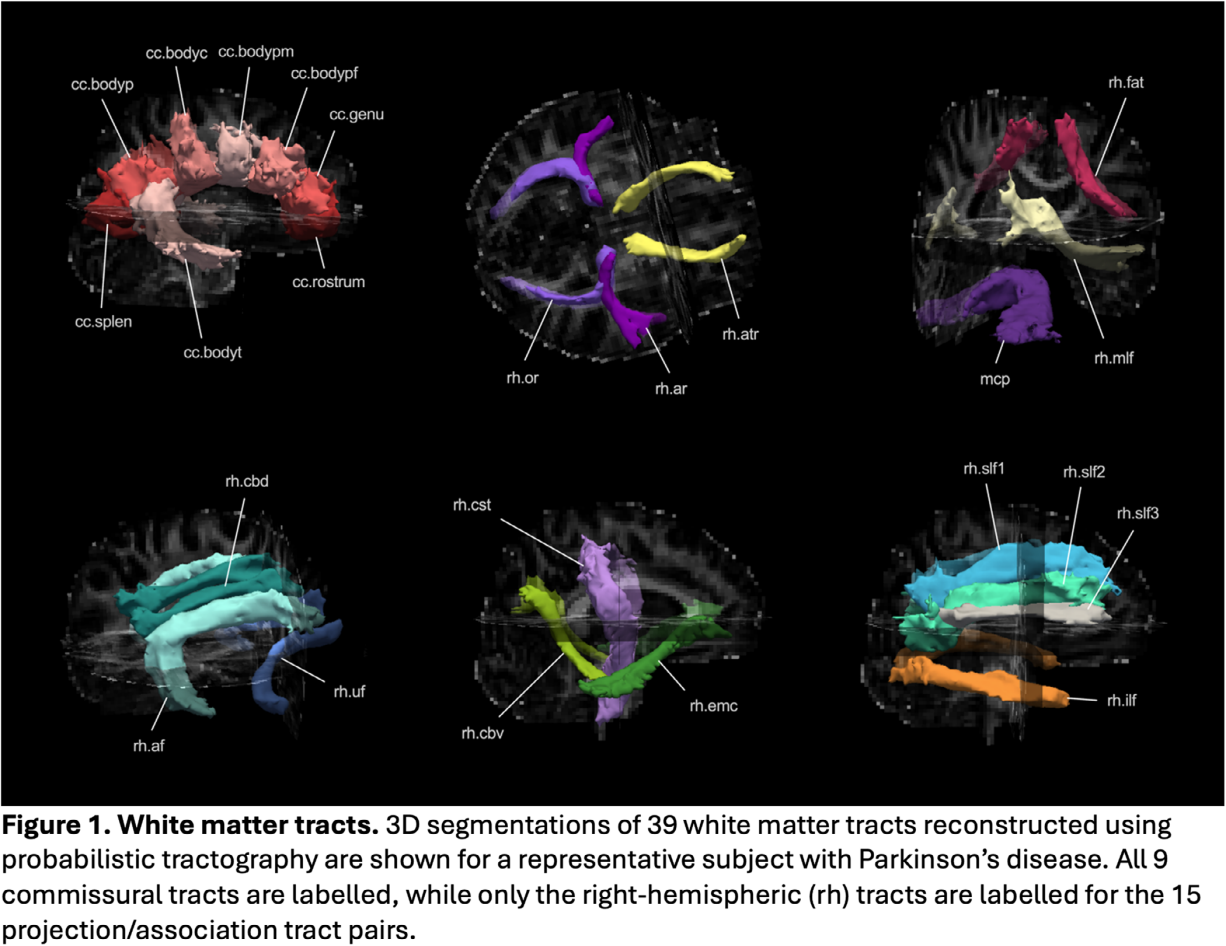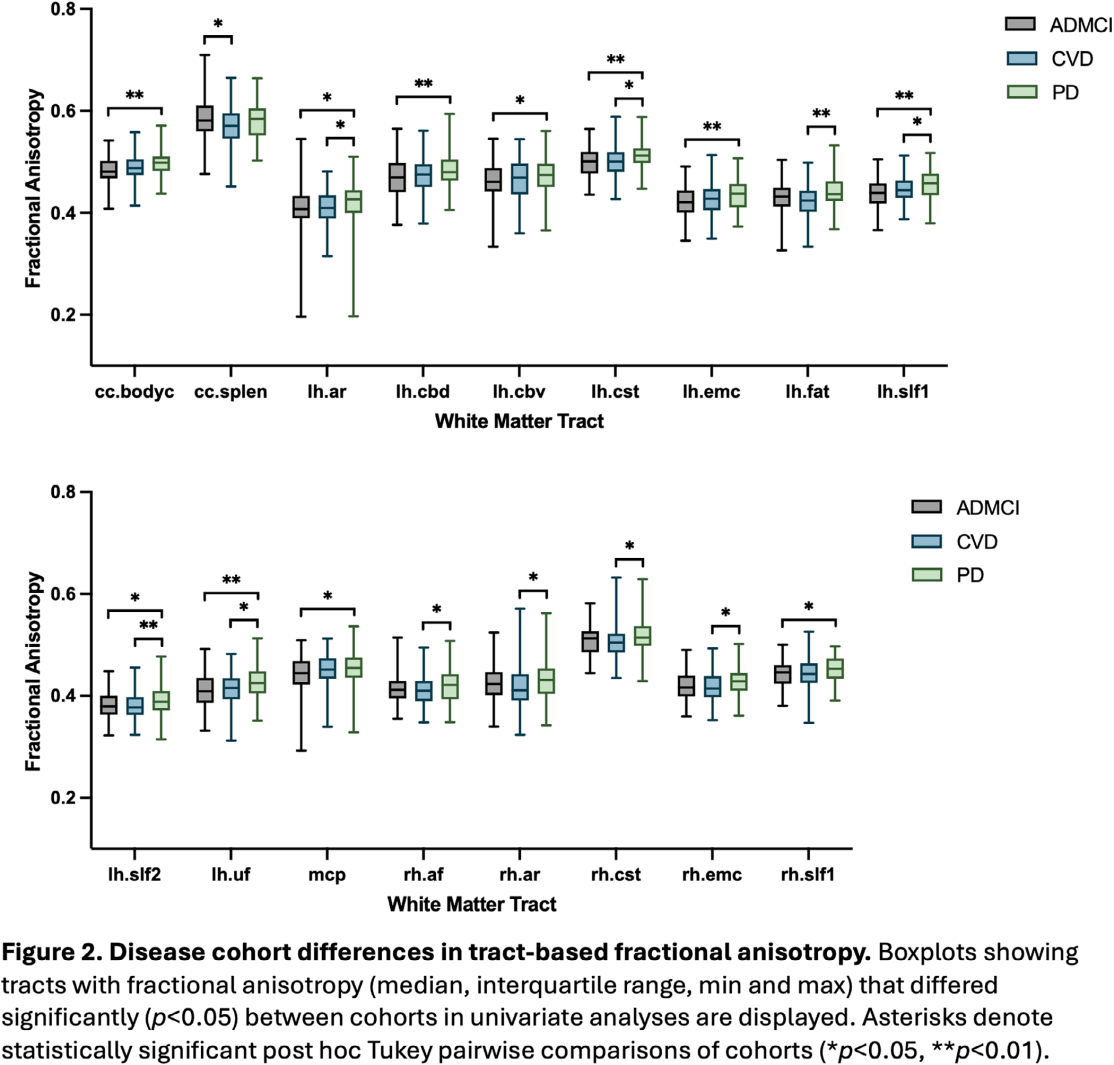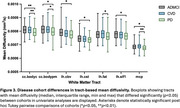# Microstructural differences in white matter tracts in Alzheimer's disease, cerebrovascular disease, and Parkinson's disease

**DOI:** 10.1002/alz70856_104478

**Published:** 2025-12-26

**Authors:** Dana N Broberg, Sandra E. Black, Richard H. Swartz, Anthony E Lang, Angela C. Roberts, Robert Bartha, ONDRI Investigators

**Affiliations:** ^1^ Robarts Research Institute, London, ON, Canada; ^2^ Western University, London, ON, Canada; ^3^ Sunnybrook Research Institute, Toronto, ON, Canada; ^4^ University of Toronto, Toronto, ON, Canada; ^5^ Toronto Western Hospital, Toronto, ON, Canada

## Abstract

**Background:**

Though previous research supports diffusion tensor imaging (DTI) as a biomarker for white matter integrity in diseases such as Alzheimer's disease/mild cognitive impairment (ADMCI), Parkinson's disease (PD), and cerebrovascular disease (CVD), few studies have compared white matter microstructure between different neurodegenerative aetiologies. This study aimed to characterize and compare the microstructure of white matter tracts in ADMCI, PD, and CVD patients using DTI data from the Ontario Neurodegenerative Disease Research Initiative (ONDRI).

**Method:**

The ONDRI study included 119 ADMCI, 149 CVD, and 137 PD patients with usable DTI and T_1_ MRI data. FreeSurfer's TRACULA [https://dmri.mgh.harvard.edu/tract‐atlas/] was used to reconstruct 39 white matter pathways and calculate average DTI metrics (fractional anisotropy, FA; mean diffusivity, MD; axial diffusivity, AxD; and radial diffusivity, RD) in each pathway. Patients with severe cerebral infarcts, hypointensities, or ventricular dilation that compromised tract reconstruction were excluded from further analyses. The final dataset included 96 ADMCI [n(%)_female_=50(52%); median(range)_age_=71(53‐87)], 107 CVD [n(%)_female_=35(33%); median(range)_age_=69(55‐85)], and 117 PD [n(%)_female_=28(24%); median(range)_age_=68(55‐84)] participants. Multivariate general linear models evaluated the effect of diagnosis on FA, MD, AxD, and RD in all 39 tracts.

**Result:**

3D segmentations of the 39 white matter tracts in a representative subject are shown in Figure 1. In the multivariate analyses, diagnosis had a strong, significant effect on all four DTI metrics (FA: *p* = 0.002, ηp^2^=0.180; MD: *p* = 0.001, ηp^2^=0.183; AxD: *p* = 0.003, ηp^2^=0.178; RD: *p* = 0.002, ηp^2^=0.181). Figures 2 and 3 display the post hoc pairwise comparisons of FA and MD, respectively, in only the tracts for which diagnosis had a significant (*p* <0.05) effect in the univariate analyses. On average, PD participants had higher FA (2.3%, ηp^2^=0.030) and lower MD (1.7%; ηp^2^=0.032), AxD (1.3%; ηp^2^=0.036), and RD (2.3%; ηp^2^=0.027) within these tracts compared to ADMCI and CVD participants. There were very few significant differences between ADMCI and CVD participants.

**Conclusion:**

PD participants had slightly more preserved white matter tract microstructural integrity than ADMCI or CVD participants who had lower FA and higher RD and MD values. Higher‐quality, advanced diffusion imaging may be necessary to detect the more subtle changes in white matter microstructure present in Parkinson's disease patients.